# Identification of *KSR2* Variants in Pediatric Patients with Severe Early-Onset Obesity from Qatar

**DOI:** 10.3390/genes15080966

**Published:** 2024-07-23

**Authors:** Lubna I. Abu-Rub, Tara Al-Barazenji, Sumaya Abiib, Ayat S Hammad, Alaa Abbas, Khalid Hussain, Mashael Al-Shafai

**Affiliations:** 1 Department of Biomedical Sciences, College of Health Sciences, QU Health, Qatar University, Doha 2713, Qatar; lubna.ali@qu.edu.qa (L.I.A.-R.); tara.b@qu.edu.qa (T.A.-B.); sumaya.abiib@qu.edu.qa (S.A.); ayat.hammad@qu.edu.qa (A.S.H.); aa1604042@student.qu.edu.qa (A.A.); 2 Division of Endocrinology, Department of Pediatric Medicine, Sidra Medicine, Doha P.O. Box 26999, Qatar; 3 Biomedical Research Center, Qatar University, Doha P.O. Box 2713, Qatar

**Keywords:** *KSR2*, childhood obesity, monogenic obesity, early-onset obesity, severe obesity, whole genome sequencing, Qatar

## Abstract

The kinase suppressor of Ras 2 (*KSR2*) gene is associated with monogenic obesity, and loss-of-function variants in *KSR2* have been identified in individuals with severe early-onset obesity. This study investigated *KSR2* variants in 9 pediatric patients with severe early-onset obesity in Qatar using whole genome sequencing among a cohort of 240 individuals. We focused on *KSR2* variants with a minor allele frequency (MAF) below 1% and a Combined Annotation Dependent Depletion (CADD) score above 13 to identify potential causative variants. Our analysis identified four *KSR2* variants: one intronic (c.1765-8G>A) and three missense variants (c.1057G>A, c.1673G>A, and c.923T>C) in nine patients. The intronic variant c.1765-8G>A was the most frequent (seen in six individuals) and had a CADD score of 21.10, suggesting possible pathogenicity. This variant showed a significantly higher allele frequency in the Qatari population compared to the Genome Aggregation Database (gnomAD), indicating a possible founder effect. Molecular modeling of the missense variants revealed structural changes in the protein structure. The study concludes that these four *KSR2* variants are associated with monogenic obesity, with an autosomal dominant inheritance pattern. The c.1765-8G>A variant’s prevalence in Qatar underscores its importance in genetic screening for severe obesity. This research advances the understanding of genetic factors in severe early-onset obesity and may inform better management strategies.

## 1. Introduction

Obesity is a global health concern with a continuously increasing prevalence (World Obesity Organization, 2023). In the Middle East, obesity prevalence has increased from 11% in 2000 to 23% in 2020 among children and adolescents, and this percentage is expected to double between 2020 and 2035 [1,2]. In Qatar, it was reported that 31.71% of boys and 32.78% of girls between the ages of 6 and 11 years were either overweight or obese [3]. In a recent study in Qatar, the percentage of students classified as overweight or obese rose from 44% in the academic year 2016–17 to 49.30% in 2019–20, accompanied by a slight decline (from 48.80% to 47.80%) in the proportion of students with a normal body mass index (BMI) [4].

Various factors, both environmental and genetic, contribute to an individual’s susceptibility to obesity. Environmental factors, such as a sedentary lifestyle and unhealthy dietary habits, are well-established contributors to the risk of obesity [5]. In addition, several studies have suggested a strong genetic influence on obesity susceptibility with an estimated heritability of 70–80% for obesity and BMI through twin studies [6]. Advances in scientific research have facilitated a deeper understanding of the genetic mechanisms that influence BMI variation [7], resulting in the classification of obesity at the genetic level into monogenic and polygenic forms [5]. Polygenic obesity is the common form of obesity caused by genetic variants conferring susceptibility to obesity in interaction with environmental factors [8]. In contrast, monogenic obesity (MO) is a rare form of obesity caused by single gene variants that lead to severe and early-onset obesity and is inherited in a Mendelian pattern [9,10,11].

MO is mainly caused by variants in genes that encode components of the leptin–melanocortin pathway in the central nervous system (CNS) [12,13]. Among these, variants in the melanocortin-4 receptor gene (*MC4R*) are the most frequently reported causes for MO accounting for approximately 5–6% of cases [14]. Other notable genes implicated in MO include leptin (*LEP*), leptin receptor (*LEPR*), carboxypeptidase E (*CPE*), pro-opiomelanocortin (*POMC*), prohormone convertase 1 (*PCSK1*), the melanocortin 3 receptor (MC*3*R), melanocortin 2 receptor accessory protein 2 (*MRAP2*), adenylyl cyclase 3 (*ADCY3*), and kinase suppressor of Ras 2 (*KSR2*) [9,12,15,16].

In this study, we focused on variants in the *KSR2* gene, which encodes a scaffolding protein that plays a role in coordinating various signaling pathways, such as the Raf/MEK/ERK signaling pathway [17]. It collaborates with *KSR1* to modulate signal transduction and regulate the strength and duration of ERK signaling [18,19]. *KSR2* also interacts with and enhances the activation of AMPK, a key regulator of cellular energy balance [20], influencing food intake, basal metabolic rate, fatty acid oxidation (FAO), and glucose oxidation in humans [19]. *KSR2* is predominantly expressed in the brain, especially in the cortex and cerebellum [17]. The reported obesity cases linked to *KSR2* variants are relatively rare and exhibit an autosomal dominant pattern of inheritance [19,21,22,23]. A notable study conducted in the UK by Pearce et al. involving 2101 individuals with severe early-onset obesity and 1536 controls identified 27 rare *KSR2* variants. The carriers of these variants exhibited hyperphagia in childhood, bradycardia, diminished basal metabolic rate, and severe insulin resistance, collectively indicating the significant role of *KSR2* in regulating energy intake, energy expenditure, and substrate utilization in humans [19]. Similarly, Körber et al. conducted a cohort study in Germany involving 88 children with a suspected diagnosis of monogenic obesity. Among them, nine patients were identified with five rare missense *KSR2* variants, each bearing in silico predictions of deleterious consequences as determined through computational prognostication tools [21]. Additionally, a recent study carried out in the USA involving 117 youth subjects with early-onset obesity identified 2 individuals with heterozygous *KSR2* variants: c.2491C>G (p.Leu831Val) and c.893C>T (p.Thr298Met) [23]. MO remains understudied in Middle Eastern populations and, to the best of our knowledge, no *KSR2* variants causing MO were reported in the literature in these populations until now. 

Research utilizing animal models has provided significant insights into the role of *KSR2* in metabolic regulation. Specifically, studies involving *KSR2* knockout mice have demonstrated the development of obesity, thereby implicating *KSR2* in the regulation of energy homeostasis [24]. Furthermore, investigations by Gomez et al. in *KSR2* knockout mice revealed a detrimental impact on femoral bone mass and bone formation, a process mediated through the modulation of adipocyte differentiation [25], suggesting an additional role of *KSR2* in bone homeostasis.

In our study, we investigated the role of *KSR2* gene variants in a cohort consisting of 240 unrelated pediatric participants with severe early-onset obesity from the population of Qatar recruited from the endocrinology clinic at Sidra Medicine to better understand the significance of *KSR2* gene variants in the etiology of obesity and the broader spectrum of metabolic regulation in such an understudied Middle Eastern population. 

## 2. Materials and Methods

### 2.1. Ethical Approval and Family Enrolment

The study was conducted in accordance with the Declaration of Helsinki and received ethical approval from the Institutional Review Boards of Sidra Medicine (IRB #1689931) and Qatar University (IRB #1568-E/21), and for Qatar Biobank/Qatar Genome data (IRB# E -2017-QF-QBB- RES-ACC-0087-0034). Recruitment of our participants (240 individuals) was conducted within the Endocrinology Division of the Pediatric Department at Sidra Medicine retrospectively and prospectively. The inclusion criteria for participants were age below 18 years, onset of obesity before 10 years, and a BMI exceeding the 99th percentile according to the criteria established by the World Health Organization (WHO) [26]. Cases attributed to secondary causes of obesity, such as syndromic obesity, were excluded from the study. Prior to participation, written informed consent was obtained from all participants’ parents. Comprehensive physical examinations, conducted at the Pediatric Endocrinology Clinic, included recording various anthropometric measures such as bioelectrical impedance analysis, height, and BMI. Additionally, 4 mL blood samples were obtained in an EDTA tube for DNA extraction.

### 2.2. Whole Genome Sequencing and Variants Analysis

Genomic DNA was extracted from the patients’ blood samples using the QIAamp DNA Blood Midi Kit (QIAGEN, Hilden, Germany; catalog # 51185). Whole genome sequencing (WGS) was conducted using the Illumina NovaSeq 6000 platform (Illumina, San Diego, CA, USA) at the Integrated Genomic Services, at Sidra Medicine, on a fee-for-service basis. Sequencing data were processed and aligned to the reference genome using Illumina’s bcl2fastq software and bwakit (v. 0.7.11), respectively. Variant calling was performed using GATK 3.4, and a multi-sample VCF file was generated. Variants marked as PASS were retained after applying the VQSR step. The annotation of variants followed GATK best practices and utilized coding DNA reference sequences. The analysis focused on MO genes incorporated in the Comprehensive Monogenic Obesity Panel from Prevention Genetics (https://www.preventiongenetics.com, accessed on 10 August 2023) and we also investigated all other genes [14,27,28,29,30,31]. Our analysis focused on variants with a minor allele frequency (MAF) below 1% and a Combined Annotation Dependent Depletion (CADD) score above 13 to identify potential rare causative variants.

### 2.3. In Silico Modelling

In silico modeling of proteins encoded by the identified *KSR2* variants was performed using the PyMOL Molecular Graphics System [32] and the Integrated Tool for Automated Structure Prediction (I-TASSER) [33]. Protein sequences were retrieved from the Universal Protein Resource (UniProt), followed by the development of 3D crystal models using the I-TASSER online tool. Evaluation of the wild-type structure was conducted using various validation tools such as ERRAT [34]. Graphics and alignment analysis between the wild-type and mutant protein structures were performed using PyMOL. Furthermore, the identified variants were searched against publicly available population databases such as gnomAD v4.0 (https://gnomad.broadinstitute.org/, accessed on 15 August 2023) and the Greater Middle East Variome (http://igm.ucsd.edu/gme/index.php, accessed on 23 August 2023). Computational pathogenic scores were obtained using CADD [35], Polymorphism Phenotyping (PolyPhen-2) (http://genetics.bwh.harvard.edu/pph2/, accessed on 2 September 2023) [36], Sorting Intolerant From Tolerant (SIFT) (https://sift.bii.a-star.edu.sg/, accessed on 2 September 2023) accessed on 14 September 2023 [37], and MutationTaster (https://www.mutationtaster.org/, accessed on 2 September 2023) [38]. 

### 2.4. Variant Searching through the Qatar Genome Program (QGP) Dataset

The Qatar Genome Program (QGP) is a national initiative to map the Qatari genome towards the implementation of precision medicine in Qatar. The whole genome sequencing dataset comprises more than 14,000 genomes from Qatar that were deeply phenotyped through the Qatar Biobank. The *KSR2* variants were searched for in the QGP dataset to determine the allele frequency, genotypes, and phenotypes of individuals with the identified *KSR2* variants. 

### 2.5. Statistical Analysis

Statistical enrichment analysis was conducted to assess the prevalence of the most reported variants across different populations. The allele counts were compared between the gnomAD dataset and the QGP data. Fisher’s exact test was used to determine if there was a significant difference in allele frequencies between these populations. *p*-values less than 0.05 were considered significant.

## 3. Case Presentations

We are presenting cases identified with rare *KSR2* variants (c.1765-8G>A, c.1057G>A, c.1673G>A, and c.923T>C) in nine patients. No other potential causative variants were identified in other genes.

Case 1

A 14-year-old Qatari male exhibited a prolonged history of morbid obesity, which began around the age of 8 to 9 years. Despite his obesity, the patient’s developmental trajectory remained unremarkable, with consistently satisfactory academic performance. His dietary regimen consisted of high-calorie foods accompanied by limited physical activity. His latest weight measurement was 104.50 kg and his height was 176.90 cm, yielding a BMI of 33.40 kg/m^2^, placing him in the 99.9th percentile with a Z-score of 2.34. The patient’s family history revealed a strong predisposition to obesity and diabetes mellitus (DM), with several members in the family having undergone sleeve gastrectomy. The patient displayed prediabetic symptoms, as evidenced by the latest hemoglobin A1c (HbA1c) level of 5.80%. He also presented with hepatic steatosis and vitamin D deficiency. Notably, thyroid-stimulating hormone (TSH) levels were elevated (7.17 mlU/L), suggesting subclinical hypothyroidism. Whole genome sequencing identified a heterozygous variant in the *KSR2* gene, c.1765-8G>A (Table 1).

Case 2

A 16-year-old Qatari female first attended the clinic at 13 years of age, weighing 99.47 kg. Her weight subsequently increased to 107.90 kg by the age of 16 and her height was 160.40 cm, resulting in a BMI of 42.10 kg/m^2^, which placed her in the 99.98th percentile with a Z-score of 2.35. The patient was diagnosed with type 2 diabetes mellitus and initially prescribed Metformin at 500 mg, which was then increased to 1000 mg. Initial attempts at weight management were marked by success through the implementation of healthy food choices and physical activity, but later she regained weight. Whole genome sequencing revealed a heterozygous variant in the *KSR2* gene, c.1765-8G>A (Table 1).

Cases 3 and 4

This case involved two Qatari female siblings suffering from severe obesity. The younger sister, aged 12 years, developed early-onset obesity at 3 years of age. By the age of 9 years, her weight had reached 72 kg. Clinical assessment indicated elevated levels of free triiodothyronine (T3), suggesting primary hypothyroidism. Her lipid profile showed elevated total cholesterol, triglycerides, and low-density lipoprotein (LDL) cholesterol levels. These levels were normalized after being prescribed Metformin, starting at 500 mg and increasing to 1000 mg. From 9 to 11 years old, her weight was maintained at 72 kg. However, upon discontinuation of Metformin at age 13 due to the patient’s non-compliance, her weight increased to 92.65 kg. Coupled with a height of 159.20 cm, this resulted in a BMI value of 36.50 kg/m^2^, which placed her in the 99.96th percentile with a Z-score of 2.68. Additionally, she started suffering from learning difficulties. Genetic analysis revealed a homozygous variant in the *KSR2* gene, c.1765-8G>A (Table 1).

The older sibling, a 13-year-old female, displayed a similar pattern of early-onset obesity beginning at the age of 3 years, accompanied by the manifestation of insulin resistance. The patient also had developmental delays and learning difficulties. Her maximum recorded weight was 114.52 kg at the age of 13 years, with a height of 156.50 cm, resulting in a BMI of 46.60 kg/m^2^, placing her in the 100th percentile with a Z-score of 2.72. Genetic testing identified a heterozygous variant, c.1765-8G>A, in the *KSR2* gene (Table 1).

Case 5

**Figure 1:**
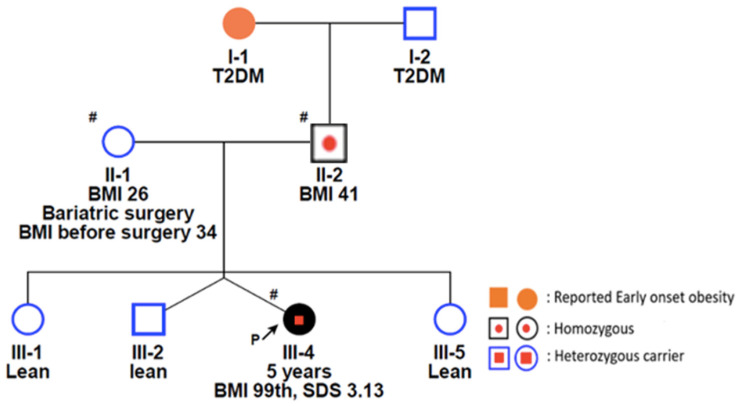
Family pedigree of Case 5 with a family history of childhood obesity. The proband and both parents were included in the family segregation. The father was homozygous for the identified variant (c.1765-8G>A) in the *KSR2* gene while the proband was heterozygous for the same variant. # Individuals who underwent genetic testing.

A 5-year-old female patient of Qatari/Saudi descent presented with early-onset obesity that initially manifested at 2 months of age. Notably, she had a twin brother who was lean and did not participate in the study. Her latest recorded weight was 36.75 kg, with a height of 115.50 cm, resulting in a BMI of 27.78 kg/m^2^, placing her above the 99th percentile and corresponding to a Z-score of 3.13. At the age of 5 years, the patient was found to have high levels of free thyroxine (T4) at 15.80 pmol/L, potentially indicative of subclinical hyperthyroidism. The patient’s family history was marked by consanguinity, as her parents were distant relatives (same tribe). The patient’s mother underwent sleeve surgery, reducing her BMI from 34 to 26 kg/m^2^, while the father demonstrated adult-onset obesity with a BMI of 41 kg/m^2^. Whole genome sequencing was performed for the patient and both parents. The sequencing results indicated that the father was homozygous for the c.1765-8G>A variant and the patient was in a heterozygous state (Figure 1).

Case 6

A 7-year-old Saudi boy presented with early-onset obesity, which began to manifest at 3 months of age. By the age of 2 years, his weight had escalated to 26 kg. Notably, his family history includes a consanguineous marriage. Genetic analysis identified a heterozygous, missense variant in the *KSR2* gene: c.1673G>A (p.Arg558Gin) (Table 1).

Case 7

A 17-year-old Qatari male with reported early-onset obesity weighed 119 kg and measured 170 cm tall, resulting in a BMI of 41.20 kg/m^2^, placing him above the 99th percentile, with a Z-score of 2.76. The patient reported experiencing polyuria for over a year, accompanied by increased thirst and fatigue in recent months. He also presented with persistent polyuria and episodes of nocturnal enuresis. Subsequent clinical assessment led to a diagnosis of type 1 diabetes mellitus, with medical records indicating the presence of diabetic ketoacidosis, requiring diabetic medications. The patient’s grandfather had diabetes, and his father had morbid obesity. Genetic testing revealed a heterozygous, missense variant, c.1057G>A (p.Val353Ile), in the *KSR2* gene (Table 1).

Case 8

**Figure 2:**
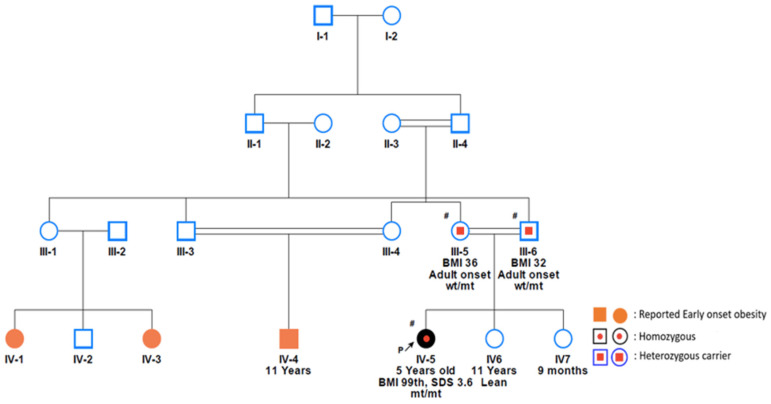
Family pedigree of Case 8 with a family history of childhood obesity. The proband and both parents have been incorporated into the family segregation analysis. Both parents were found to be heterozygous for the c.1765-8G>A variant, while the proband was found to be homozygous for the identified variant. # Individuals who underwent genetic testing.

A 4-year-old Qatari female was born with an initial weight of 5 kg and subsequently developed severe early-onset obesity, reaching 34 kg at 2 years of age. By age 4, her weight increased to 44.40 kg with a height of 116 cm, resulting in a BMI of 32.30 kg/m², placing her above the 99th percentile and corresponding to a Z-score of 3.6. Her clinical manifestations included hyperphagia, acanthosis nigricans, delayed motor skills development, and speech delay. Additionally, her clinical assessment indicated high levels of free T4, possibly indicating hyperthyroidism and elevated LDL cholesterol levels. The family had a history of consanguinity and a phenotype of early-onset obesity in several family members (Figure 2). The father and mother had a BMI of 32 kg/m^2^ and 36 kg/m^2^, respectively. Whole genome sequencing was performed for the patient and her parents. The patient was found to be homozygous for the c.1765-8G>A variant in the *KSR2* gene, while both parents were heterozygous for the same variant (Figure 2 and Table 1).

Case 9

A 5-year-old Qatari female was diagnosed with early-onset obesity at 12 months of age. Her latest recorded weight was 32.50 kg, her height was 118.10 cm, and her BMI was 23.30 kg/m^2^, which was above the 99th percentile and corresponded to a Z-score of 2.66. Clinically, the patient exhibited mild acanthosis nigricans on her neck and lipomastia of the chest. A review of her family history revealed a prevalence of obesity and type 2 diabetes. Genetic testing revealed a heterozygous, missense variant, c.923T>C (p.Leu308Ser), in the *KSR2* gene (Table 1).

## 4. Variants Analysis

The c.1765-8G>A, c.923T>C, and c.1673G>A variants had high CADD scores (>20), indicating their potential pathogenicity. The three missense variants, c.1673G>A, c.1057G>A, and c.923T>C, had conflicting pathogenicity predictions through the different in silico tools, PolyPhen-2, SIFT, CADD, and MutationTaster (Table 2). Furthermore, their allele frequencies were found to be less than 1% in gnomAD and they were not reported in GME. Notably, the c.1765-8G>A variant was never reported in the homozygous state in gnomAD, while we detected it in the homozygous state in two subjects in our study cohort. All variants were not found in the QGP database except for the c.1765-8G>A variant, which had an allele frequency of 0.00907 (Table 2).

### 4.1. Molecular Visualization Analysis

**Figure 3:**
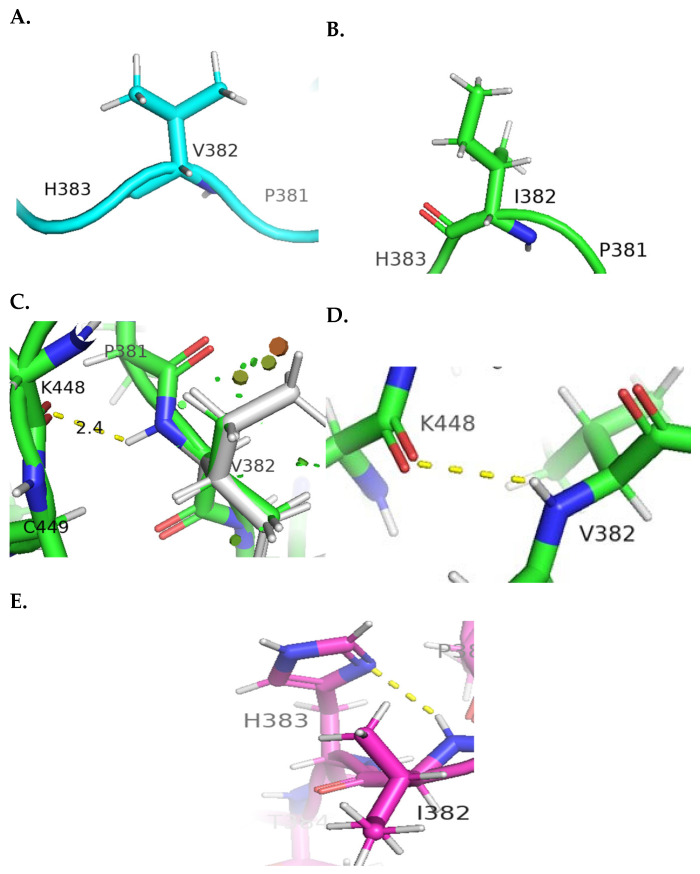
**Molecular visualization of the c.1057G>A variant.** (**A**) The wild-type amino acid (V382) in the *KSR2* protein. (**B**) The mutated amino acid (I382). (**C**) In the wild-type mutagenesis protein, minor clash points found with neighboring residues are shown as small green discs, while the bigger discs represent major clash points. (**D**) Polar contacts formed between the wild-type V382 residue and K448. (**E**) In the mutant protein, the polar contacts shift between I382 and H383.

Molecular visualization analysis of the identified variants was performed utilizing the PyMOL software (version 2.5.7). Selection of the wild-type (WT) protein model was based on the protein C-score criteria (−0.85). The quality of the predicted wild-type model was verified using the ERRAT tool, yielding a score of 82.79%, which is very satisfactory. Additionally, the ProSA prediction tool produced a Z-score of −6.93, which fell within the acceptable range for model validation (−10 to 10) [39]. Visualization of the molecular changes after mutagenesis of the WT *KSR2* protein model was achieved using the PyMOL software. The variant c.1057G>A, which has transcript number NM_173598.6 and matches the published variant (c.1879G>A), resulted in a substitution of the non-polar amino acid valine at position 382 with the non-polar amino acid isoleucine which has an extra methyl group, leading to both minor and significant steric clashes with the surrounding residues and increased polar affinity to the adjacent amino acid lysine 448 (K448) (Figure 3C,D). 

**Figure 4:**
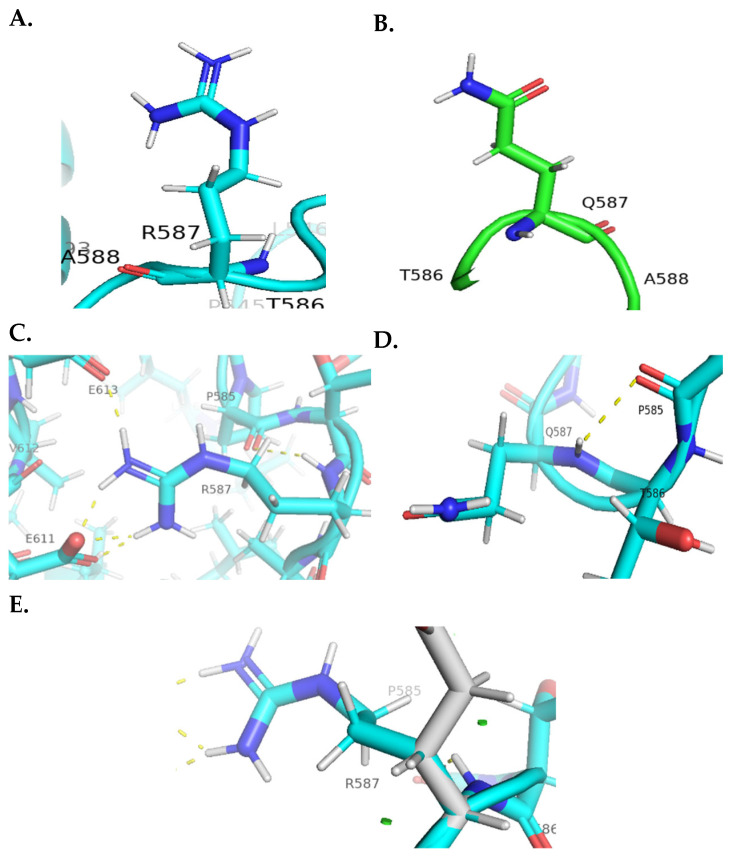
**Molecular visualization of the c.1673G>A variant.** (**A**) The wild-type amino acid (R587) in the *KSR2* protein. (**B**) The mutant amino acid (Q587). (**C**) Polar contacts of the WT (R587) residue with the adjacent amino acids, E611 and E613. (**D**) Polar contacts of the mutant residue (Q587) with the adjacent amino acid, P585. (**E**) Minor clash points found with neighboring residues are shown as small green discs in the WT protein.

Moreover, the c.1673G>A variant, which has transcript number NM_173598.6 that matches the published variant (c.2495G>A), resulted in a change in the positively charged arginine residue at position 587 to the charge-neutral, polar glutamine residue, revealing minor clashes with the surrounding molecules after mutagenesis, along with a loss of polar contacts to the adjacent amino acids Glu613 (E613) and Glu611 (E611) and gain of a new polar contact with the non-polar amino acid proline (P585), potentially influencing the protein’s function or stability (Figure 4C–E). 

**Figure 5:**
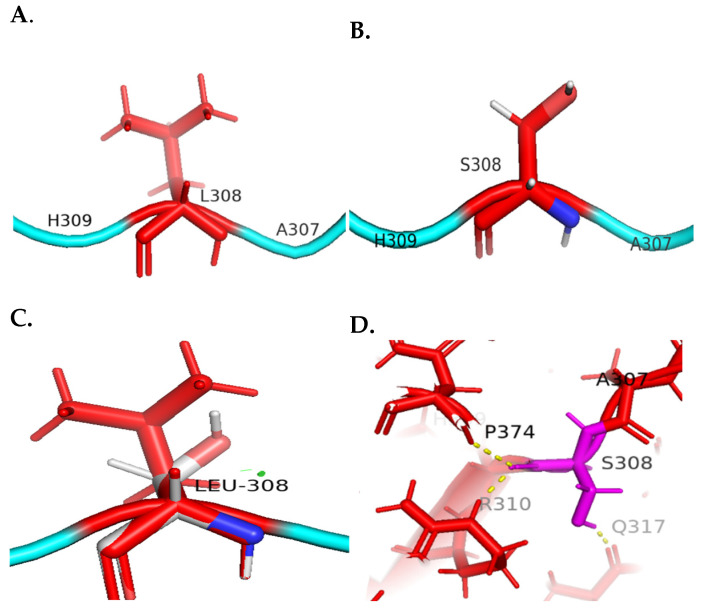
**Molecular visualization of the c.923T>C variant in *KSR2*.** (**A**) The wild-type amino acid (L308) in the *KSR2* protein. (**B**) The mutant amino acid, S308. (**C**) Mutagenesis showed minor clash points found with neighboring residues shown as small green discs in the WT protein. (**D**) Polar contacts of the mutant S308 residue with the adjacent amino acids P374, R310, and Q317.

Furthermore, the c.923T>C variant resulted in an amino acid substitution at position 308, where the non-polar residue leucine was replaced by the polar residue serine. This substitution engendered localized structural perturbations, as evidenced by the minor steric clashes with proximate amino acid residues (Figure 5C). Furthermore, the introduction of the polar serine residue at this site enhanced polar interaction with adjacent amino acids, specifically proline at position 374, arginine at position 310, and glutamine at position 317 (Figure 5D). These alterations in inter-residual interactions consequent to the leucine to serine substitution have a notable impact on the overall stability of the protein’s tertiary structure. The increased polar affinity introduced by the serine residue at position 308 appears to disrupt the pre-existing non-polar interactions within the protein’s interior, thereby potentially destabilizing the native conformational state of the protein. 

**Figure 6:**
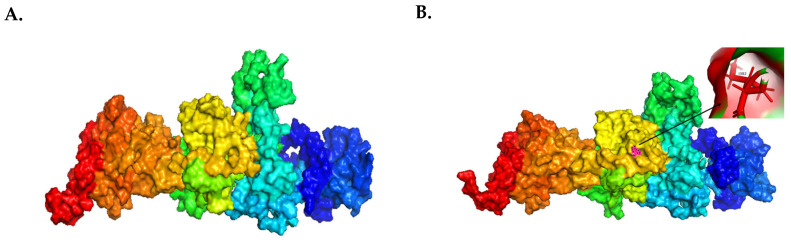

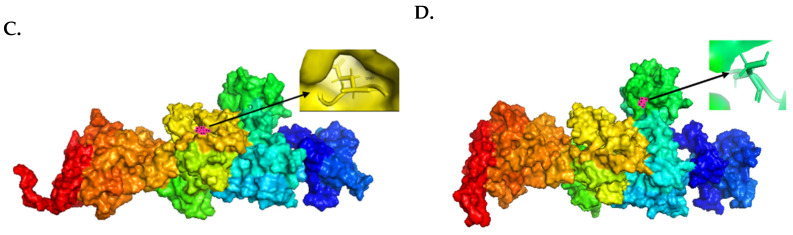
**Predicted protein models of the WT and other mutant proteins**. (**A**) Surface protein model of the wild-type *KSR2* protein. Surface protein models of the mutant types are shown for (**B**) I382, (**C**) Q587, and (**D**) S308. The arrows point to the mutated amino acids, which are displayed in a stick model.

The root mean square deviation (RMSD), a critical metric for assessing structural deviations, was calculated using the PyMOL prediction tool by performing an alignment analysis between the wild-type protein structure (Figure 6A) against the three mutant protein models for the three *KSR2* variants (I382, Q587, and S308). The RMSD values for the mutant types S308 and Q587 were 7.698 and 7.325, respectively. These values, being relatively high, indicate a notable degree of misalignment when these mutant structures are compared to the wild-type conformation (Figure 6C,D). Conversely, the RMSD value for the I382 mutant type was 2.41, which might be considered low, suggesting that the structures were relatively similar, with only minor differences (Figure 6B).

### 4.2. Enrichment Analysis

Within the gnomAD dataset, the allele count for the intronic variant, c.1765-8G>A, was found to be 8. However, analysis of the QGP data revealed a significantly elevated allele count of 261 for the same variant. This suggests that this variant is much more prevalent in the Qatari population when compared to the larger, more diverse population represented by gnomAD. Statistical analysis using Fisher’s exact test confirmed the enrichment of the c.1765-8G>A variant in the Qatari population compared to gnomAD with a *p*-value of *p* < 0.001 (Table 3). It is worth noting that no homozygous individuals were reported for this variant in gnomAD, while we identified two homozygous Qatari subjects in our cohort. 

Further investigation of this variant (c.1765-8G>A) conducted through the QGP database revealed 11 individuals documented as homozygous and 238 individuals documented as heterozygous. Interestingly, individuals from both the heterozygous and homozygous categories exhibit a range of body mass indices, including lean, overweight, and obese individuals. However, obese individuals are present at the highest frequency within both zygosity groups (Table 4).

## 5. Discussion

This study conducted an analysis of nine Arab patients in Qatar with early-onset obesity with variants in the *KSR2* gene. The investigation identified four rare variants (c.1765-8G>A, c.1057G>A, c.1673G>A, and c.923T>C) in the *KSR2* gene. The gene encodes a scaffolding protein of 950 amino acids predominantly expressed in the brain, which regulates different signaling pathways. The initial association between *KSR2* genetic variants and severe early-onset obesity was established by Pearce et al. in the United Kingdom [19]. Subsequent research, including various studies and case reports, has further explored the relationship between *KSR2* gene variants and obesity or related metabolic conditions in both human and animal models. These studies have significantly enhanced our understanding of the genetic factors influencing energy balance, metabolism, and obesity [17,20,22,23,25,40].

To the best of our knowledge, our study is the first to report these variants in the *KSR2* gene (c.1765-8G>A, c.1673G>A, c.1057G>A, and c.923T>C), classified as VUSs, in association with early-onset obesity in the Arab population, specifically from Qatar and Saudi Arabia. According to the gnomAD database, these variants are rare, with a frequency of less than 1%, and have not been previously reported in the GME. Although the identified variants were previously reported in publicly available databases, this is the first time they are discussed in the context of severe early-onset obesity, suggesting their potential pathogenic role in the development of this condition and highlighting the need for functional studies to enable their reclassification. 

The most prevalent variant in our patients was the intronic variant (c.1765-8G>A), which was detected in six Qatari patients (two homozygous and four heterozygous). The variant was enriched in the Qatari population, represented by the QGP database, compared to the gnomAD population, which may be due to the founder effect. The c.1765-8G>A variant had a high CADD score of 22.60, indicating a significant potential for pathogenicity possibly by disrupting normal splicing processes and leading to functional consequences. Thus, this variant warrants further investigation due to its potential impact on protein structure and function and its frequent association with early-onset obesity in our study cohort. Notably, intronic variants can result in monogenic diseases by various mechanisms, including disruption of splicing branch points or splicing canonical sites, pseudo-exon activation, and poison exon inclusion [41]. 

Significantly, the c.1765-8G>A variant was detected in both heterozygous and homozygous states in two different individuals (Cases 5 and 8) belonging to the same tribe. This genetic variant was identified in the QGP database and displays diverse phenotypic expressions across individuals and families. Notably, individuals with both homozygous and heterozygous states for this variant exhibit a spectrum of phenotypes, ranging from lean to obese. 

Examining the segregation of this variant across different families reveals significant variability in phenotypic outcomes. In Case 5, for instance, the patient presented with early-onset obesity due to heterozygosity for the variant, while the father, homozygous for the same variant, experienced adult-onset obesity. This disparity highlights the concept of variable expressivity, where the same genetic variant can lead to different phenotypic manifestations even within a single family. 

Another illustrative case is Case 8, where the severity of the observed phenotype correlates with the zygosity of the genetic variant. Both heterozygous parents reported adult-onset obesity, indicating a milder manifestation of the condition. Conversely, the homozygous patient, carrying two copies of the variant, suffered from a more severe form of early-onset obesity. This may suggest that the zygosity of the genetic variant can contribute to the age of onset and severity of the obesity phenotype within affected families. 

Consequently, the variability in phenotypic outcomes associated with the same genetic variant underscores the complex relationship between genetic factors and resulting clinical presentations. Understanding variable expressivity and considering factors such as zygosity and environmental factor contributions are essential in deciphering the full spectrum of clinical implications associated with genetic variants [10]. 

Similar findings were reported in a previous study conducted by Pearce [19]; they reported that similar *KSR2* variants were identified in both lean and obese patients, in both the homozygous and heterozygous states, further emphasizing the variable expressivity of *KSR2* genetic variants. 

Further studies, including in vitro and/or in vivo functional assays, are necessary to fully understand the implications of this intronic variant and its role in obesity pathogenesis.

The c.1057G>A and c.923T>C variants were detected in two other Qatari patients, while the c.1673G>A variant was found in a Saudi patient with severe early-onset obesity. In silico pathogenicity predictions yielded conflicting results for both the c.1673G>A and c.1057G>A variants, whereby SIFT and PolyPhen-2 indicated that they had tolerated effects on the protein structure, whereas MutationTaster and their CADD scores (21.8 and 14.81, respectively) pointed towards their possibly damaging effects on the protein structure. Moreover, in silico predictions of the c.923T>C variant showed a tolerated effect according to SIFT and a damaging effect on the protein structure according to PolyPhen-2, CADD, and MutationTaster. Furthermore, Pearce et al.’s and Roberts et al.’s studies evidenced that the majority of the documented instances involving *KSR2* variants were characterized by an autosomal dominant inheritance pattern [19,23]. In alignment with these findings, our investigation revealed an autosomal dominant inheritance pattern in 77.8% of the cases examined. While two of the nine probands were homozygous for the intronic variant, we did not observe a consistent effect of the zygosity on BMI or age of obesity onset among the different cases.

Notably, out of the nine cases, five had either diabetes mellitus themselves or a family member with the condition. These findings align with the results of Pearce et al., where individuals with the variant showed pronounced insulin resistance. The authors discussed how some of the *KSR2* variant carriers were given metformin, which triggered AMPK activation, leading to significant weight loss in these carriers. This is consistent with the outcomes observed in Cases 2 and 3, where the administration of metformin correlated with weight loss, while its absence was associated with weight gain. All results confirm *KSR2*’s crucial role in regulating energy intake, energy expenditure, and substrate utilization in humans [19]. 

Among the cohort of nine cases, four exhibited thyroid gland abnormalities. As previously elucidated, *KSR2* assumes the role of a coordinator for diverse signaling pathways, which notably includes the Raf/MEK/ERK signaling cascade [18,19]. This intricate signaling network constitutes an essential part of cellular biology, wherein perturbations thereof have been implicated in the initiation of malignancies and developmental anomalies [42]. 

## 6. Conclusions

In this study, we have identified four genetic variants in the *KSR2* gene that are linked to early-onset monogenic obesity in our cohort of seven Qatari, one Saudi, and one Qatari–Saudi patients. Although these variants were detected in publicly available databases, to our knowledge this is the first time they are discussed in the context of severe early-onset obesity. The c.1765-8G>A intronic variant was the most common variant in our cohort, detected in six cases, and was found to be enriched in the Qatari population. 

Our findings suggest that among this population, *KSR2* variants may contribute to an autosomal dominant form of early-onset monogenic obesity. Nevertheless, two of our cases (Cases 3 and 8) were homozygous for the c.1765-8G>A intronic *KSR2* variant, a significant finding that we have reported here for the first time.

Importantly, functional investigations are needed to better understand the role of the identified variants in obesity pathogenesis. Furthermore, we underscore the importance of screening for the c.1765-8G>A variant in clinical settings for patients with severe early-onset obesity, due to its enrichment in the Qatari population. This study provides new insights into the role of *KSR2* variants in the development of monogenic obesity in the understudied Qatari population and paves the way for further research in the area. 

## Figures and Tables

**Table 1:** Concise summary of the study subjects with severe early-onset obesity and the identified variants in the *KSR2* gene.

Case No.	Age at Recruitment	Gender *	Nationality	Consanguineous Marriage	BMI (kg/m^2^)	BMI Percentile	Z-Score	Age of Obesity Onset	Zygosity **	Nucleotide Change	Protein Change
1	14	M	Qatari	NR	33.40	99.90	2.34	8 years	Het	c.1765-8G>A	-
2	16	F	Qatari	NR	42.10	99.98	2.35	NR	Het	c.1765-8G>A	-
3	12	F	Qatari	NR	42.10	99.96	2.68	3 years	Hom	c.1765-8G>A	-
4	13	F	Qatari	NR	46.60	100.00	2.72	3 years	Het	c.1765-8G>A	-
5	5	F	Saudi/Qatari	Yes	27.90	100.00	3.13	2 months	Het	c.1765-8G>A	-
6	7	M	Saudi	Yes	NR	<99	NR	3 months	Het	c.1673G>A	p.Arg558Gin
7	17	M	Qatari	NR	41.20	99.66	2.83	NR	Het	c.1057G>A	p.Val353lle
8	4	F	Qatari	Yes	32.32	<99	3.60	2 months	Hom	c.1765-8G>A	-
9	5	F	Qatari	NR	23.30	<99	2.66	2 years	Het	c.923T>C	p.Leu308Ser

* F = Female and M = Male, ** Het = heterozygous, and Hom = homozygous. NR, not reported.

**Table 2:** *KSR2* variants identified in patients with severe early-onset obesity in our cohort.

Transcript	Nucleotide Change	Amino Acid Change	SIFT	PolyPhen-2	MutationTaster	CADD	ACMG Class	gnomAD	GME	QGP
NM_173598.6	c.1765-8G>A rs375107117	-	-	-	-	21.10	-	0.00007752	NR	0.00907
NM_173598.6	c.1057G>A rs1485095679	p.V382I	0.16 tolerated	0.22 benign	Disease-causing	14.81	VUS (PM1, PM2; warm)	0.0033	NR	NR
NM_173598.6	c.1673G>A rs768799651	p.R587Q	0.59 tolerated	0.02 benign	Disease-causing	21.80	VUS (PM1, PM2; warm)	0.0098	NR	NR
NM_173598.4	c.923T>C ClinVar: 2632205	p.L308S	0.07 tolerated	0.72 possibly damaging	Disease-causing	24.30	VUS (PM1, PM2; warm)	0.0039	NR	NR

VUS, variant of unknown significance; NR, not reported; ACMG, American College of Medical Genetics and Genomics.

**Table 3:** Statistical analysis results for the enrichment analysis of the c.1765-8G>A variant (rs375107117).

	gnomAD AC	QGP AC	Marginal Row Totals
Variant AC	8	261	269
WT AC	152,304	28,513	180,817
Marginal column total	152,312	28,774	181,088

The Fisher’s exact test *p*-value is *p* < 0.001. AC, allele count; WT, wild type.

**Table 4:** Distribution of body mass indices across zygosity groups for the c.1765-8G>A variant.

Zygosity	Underweight	Lean	Overweight	Obese
**Homozygous**	-	1	3	7
**Heterozygous**	15	40	81	102

## Data Availability

All newly generated data supporting the findings of this study are included within the paper. However, individual participant data are not publicly available to protect privacy and adhere to ethical guidelines.
